# Quality by Design Approach for the Development of Liposome Carrying Ghrelin for Intranasal Administration

**DOI:** 10.3390/pharmaceutics13050686

**Published:** 2021-05-10

**Authors:** Cecília de Barros, Norberto Aranha, Patrícia Severino, Eliana B. Souto, Aleksandra Zielińska, André Lopes, Alessandra Rios, Fernando Batain, Kessi Crescencio, Marco Chaud, Thais Alves

**Affiliations:** 1 Laboratory of Biomaterials and Nanotechnology (LaBNUS), University of Sorocaba, Sorocaba 18078-005, São Paulo, Brazil; cecilia.barros@edu.uniso.br (C.d.B.); alessandra.rios@edu.uniso.br (A.R.); fernando.batain@uniso.br (F.B.); kessicrescencio@yahoo.com.br (K.C.); 2 Technological and Environmental Processes, University of Sorocaba, Sorocaba 18078-005, São Paulo, Brazil; norberto.aranha@prof.uniso.br; 3 Nanomedicine and Nanotechnology Laboratory (LNMed), Institute of Technology and Research (ITP), Av. Murilo Dantas, 300, Aracaju 49010-390, Sergipe, Brazil; pattypharma@gmail.br; 4 Department of Pharmaceutical Technology, Faculty of Pharmacy, University of Coimbra, Ciências da Saúde, 3000-548 Coimbra, Portugal; ebsouto@ff.uc.pt; 5 Institute of Human Genetics, Polish Academy of Sciences, Strzeszynska 32, 60-479 Poznan, Poland; zielinska-aleksandra@wp.pl; 6 Faculty of Pharmaceutical Science, University of Campinas, Campinas 13083-871, São Paulo, Brazil; amlopes@unicamp.br; 7 College of Bioprocess and Biotechnology Engineering, University of Sorocaba, Sorocaba 18023-000, Sâo Paulo, Brazil; 8 Technological Innovation Agency of Sorocaba, Sorocaba Technology Park, Itavuvu Avenue, Sorocaba 18078-005, São Paulo, Brazil

**Keywords:** ghrelin, intranasal rout, liposomes, quality by design (QbD), cachexia, undernourishment, starvation

## Abstract

The therapeutic use of peptides has increasingly recognized in the development of new therapies. However, the susceptible enzymatic cleavage is a barrier that needs to overcome. Nose-to-brain delivery associated with liposomes can protect peptides against biodegradation and improve the accessibility to brain targets. The aim was to develop a liposomal formulation as ghrelin carrier. The quality by design (QbD) approach was used as a strategy for method development. The initial risk assessments were carried out using a fishbone diagram. A screening design study was performed for the critical material attributes/critical process parameters (CMAs/CPPs) on critical quality attributes (CQAs). Liposomes were obtained by hydrating phospholipid films, followed by extrusion or homogenization, and coated with chitosan. The optimized liposome formulation was produced by high-pressure homogenization coated with chitosan, and the resulted were liposomes size 72.25 ± 1.46 nm, PDI of 0.300 ± 0.027, the zeta potential of 50.3 ± 1.46 mV, and encapsulation efficiency of 53.2%. Moreover, chitosan coating improved performance in ex vivo permeation and mucoadhesion analyzes when compared to the uncoated liposome. In this context, chitosan coating is essential for the performance of the formulations in the ex vivo permeation and mucoadhesion analyzes. The intranasal administration of ghrelin liposomes coated with chitosan offers an innovative opportunity to treat cachexia.

## 1. Introduction

Cachexia is described in association with many chronic conditions and infectious diseases and seen in patients after extensive traumatic injuries or sepsis. Moreover, the cachexia is a devastating complication of cancer and strikes more than 50% of patients with different cancers and is responsible for the death of a minimum of 20% of all patients [1,2]. It is characterized by continuous loss of muscle mass that leads to progressive functional impairment, while nutritional support is unable to completely reverse this clinical condition [2,3].

Ghrelin (ghrl) is a 28-amino acid hormone produced primarily in the oxyntic mucosa of the stomach. This hormone binds to growth hormone secretagogue receptor the type 1a (GHS-R1a) in the hypothalamus and stimulates the release of growth hormones after catalyzed by ghrelin-O-acyltransferase (GOAT) [4]. The ghrelin GOAT system is responsible for regulating energy homeostasis, signaling the hypothalamus’ peripheral nutritional status inducing energy compensations; however, in the body, exogenous ghrelin is subject to very rapid degradation [5,6,7]. Emulsion-based nanoparticles as the liposome are reported as an alternative to avoid enzymatic hydrolysis [8].

Bilayer nanoliposomes are versatile nanostructure, biocompatible, easily functionalized for targeted drug delivery, and improve permeation and pharmacokinetics [9,10]. The incorporation of peptides into liposomes, associated with the choice of an alternative route of administration can offer several advantages in therapies using peptides [11]. The nasal route has advantages for cerebral release and chronic administration of macromolecules [12]. The olfactory region of the nasal mucosa provides a connection between the nose and the brain, which can be used to more easily distribute drugs that act on the central nervous system [13]. In this context, the intranasal release of liposomes loaded with ghrelin to cross the blood-brain barrier and the release of the drug in the central nervous system can be a promising alternative to improve its bioavailability [4].

Grafted liposomes associated with the nose-to-brain administration route improves ghrelin transmucosal permeation for targeting and increasing ghrelin protection against enzymatic biodegradation, pH, and ciliary clearance, which is promising in the treatment of cachexia [14].

Due to inadequate bioavailability through non-injection routes, the peptide therapy by parenteral route has been chosen as the first choice [15]. When chronic administrations are necessary, and considering that invasive routes are more expensive and lead to low patient compliance and consequent treatment failure, the intranasal route has advantages of being a non-invasive and painless way for drug administration [16]. The barriers of the nose-to-brain route for drug delivery are well known, and the most important ones are enzymatic and immune defense system, pH, and fast mucociliary clearance [17,18,19,20].

The quality by design (QbD) strategy was used to find the best conditions to develop liposomes for nose-brain delivery. QbD is a systematic approach to product development, starting with defined objectives and emphasizing the product, understanding the processes and control of those processes [17]. In the development of a new product, it is important to consider the therapeutic objectives, so that the research activity becomes more effective and adaptable even its initial stages. A better understanding of the product and the process facilitates the identification and control of the factors that influence the final quality of the medicine [18]. So, the QbD strategy was used to find the best conditions for liposomal formulation for nose-brain delivery, define the target profile, select the critical factors, carry out a risk assessment, and perform factorial planning. The optimized formulations of grafted chitosan were evaluated for ghrelin encapsulation efficiency, size, shape, zeta-potential, cryo-transmission electron microscopy, mucoadhesion, and ex vivo transmucosal permeation.

## 2. Materials and Methods

### 2.1. Materials

Ghrelin (obtained from rat, consisting of 28 amino acids and its residue Ser3 is N- octanilate; purity ≥ 97%) was purchased from Sigma-Aldrich, USA. Soybean phosphatidylcholine (purity ≥ 94%, LS100^®^) was obtained from Lipoid Kosmetik AG (Naroda, Steinhausen, Switzerland). Sodium salt of 1,2-diestearoyl-sn-glicero-3-phosphoethanolamine-N-[carboxi-(polyethylene glycol) 2000 (DSPE-PEG2000) was obtained from Lipoid Kosmetik AG (Naroda, Steinhausen, Switzerland). Cholesterol NF was obtained from Dishman Group (India). Ethanol PA 99%, and phosphate buffer pH 5.9—isosmotic, containing dibasic sodium phosphate P.A. (Na_2_HPO_4_) were obtained from Anidrol Produtos para Laboratórios Ltda (São Paulo., Brazil). Monobasic sodium phosphate P.A, and sodium chloride P.A. (NaCl) were obtained from Labsynth (Campinas., Brazil). Low molecular weight chitosan (75–85% deacetylated, 50,000–190,000 Da) was obtained from Sigma-Aldrich (Darmstadt, Germany). Polycarbonate membranes with pore diameters of 50 and 100 nm were purchased from Avanti Polar Lipids (Alabaster, AL, USA).

### 2.2. Liposome Preparation

The phospholipid film rehydration production method [19] was selected for the preparation of liposomes. Briefly, LS100, DSPE-PEG2000, and cholesterol, in the proportion 100:6:8% (*w*/*w*), were solubilized in ethanol under agitation for 10 min.

After solubilization, the mixture was transferred to a round-bottomed balloon. With the aid of the rotary evaporator (TE-211-Tecnal, Piracicaba, Brazil), the ethanol was evaporated at a temperature of 45 °C with pressure regulated to 300 mmHg for 20 min, or until the formation of the dry lipid film adhered to the surface of the flask was ensured. Then, the lipid film was hydrated with 10mL of phosphate buffered saline, isosmotic (PBS—pH 5.9, 308 mOsm/L) containing different ghrelin concentrations (70, 105, or 140 µg.mL^−1^). The obtained liposome suspensions were stored under refrigeration at 5–8 °C. Extrusion and high-pressure homogenization methods of post-formation processing was used for downsizing liposomes containing ghrelin.

With the aid of the Design Expert^®^ software version 12.0.0 by Stat-Ease, Inc. (Suite 480, Minneapolis, MN, USA), the balance between the categorical variables was constrained, ensuring that each post-formation processing received an equal number of runs in the experimental design.

The group of formulations selected for post extrusion processing was extruded through polycarbonate membranes. The large multilamellar liposomes (diameter ranging from 200 to 1000 nm) were extruded through the extruder with a support/heating block (610000-1EA—Avanti Polar Lipids, Alabaster, AL, USA). The holder/heating block was kept at 70 ± 5 °C, despite the high temperature the contact time is short. The formulations subsequently passed in both directions three times on each membrane with 0.1 µm porosity (EMD Millipore).

The group of formulations selected for high pressure homogenization post-formation processing (Homolab 2.20—FBF ITALIA Parma Italy) was homogenized with a double stage. The initial liposome suspensions were processed with a high-pressure homogenizer without the recirculation mode. The inlet pressure of the homogenizer was adjusted to 1000 bar and 3 cycles were performed (number of times samples were processed).

#### Chitosan-Coated Liposome

Three different chitosan concentrations (1, 3, and 5 mg. mL^−1^) were dissolved in 0.1 N acetic acid were prepared to prepare the chitosan solution. The dispersion was shaken for 4 h to obtain the final homogeneous and translucent solution. Then, 9 mL of the liposome suspension were coated with 1 mL of the chitosan dispersion to which the chitosan suspensions were added drop by drop, under constant stirring, to obtain a dilution 10 times greater than the initial chitosan dispersion. The dispersions of coated liposomes were left under constant agitation at different times (2, 8, and 24 h) and then stored at 5–8 °C.

### 2.3. Quality by Desing: Optimizing the Formulation

In this study, as a characteristic procedure of QbD, we defined the quality target product profile (QTPP); we identified the critical material attributes (CMAs) and critical process parameters (CPPs) and, consequently, the critical quality attributes (CQAs). For quality control in the development of liposomes loaded with peptides we used the tools of the initial risk assessment, design of experiments (DoE), and process analytical techniques (PATs) [17,20].

As a starting point of the QbD approach, the QTPP of the liposomal product and its quality criteria were defined as a starting point of the QbD approaches. To achieve the QTPP, the factors necessary the target product were searched in the literature and evaluated. Meanwhile the selection of factors, which have critical effects on the quality of the target product, was based on previous knowledge and experience.

CMAs and CPPs are the factors related to the materials and the selected production methods and processes, respectively, were identified using the cause-and-effect diagram (Ishikawa), moreover this diagram was also used for risk assessment, thus selecting the influential CQAs.

DoE was used in the subsequent step as a QbD tool, and the design used involves one categorical factor and two numerical factors. The numerical factors were adjusted at levels -1 (low), 0 (medium), and +1 (high), within the appropriate range determined in the initial risk assessment with 2 central point repetitions Thus, an experimental design was constructed to study the influence of CMAs and CPPs on CQAs properties. DoE was developed using the software Design Expert^®^ version 12.0.0 by Stat-Ease, Inc. (Suite 480, Minneapolis, MN, USA).

### 2.4. Liposome Characterization

#### 2.4.1. Particle Size, Polydispersion Index (PDI), and Zeta Potential

Particle size, polydispersion index (PDI), and zeta potential were determined using a particle analyzer (ZetaPALS, model NanoBrook 90PlusPALS, Brookhaven Instruments, Holtsville, NY, USA). Dynamic light scattering (DLS) was used for particle size determination and phase analysis light scattering (PALS) for surface load determination. Processed samples were further diluted in ultrapure water (1:30), and homogenized. The measurements were performed at a temperature of 25 °C, a light scattering angle of 90° and zeta potential at 15°. The particle analyzer allows the measurement of the particle size, zeta potential of the sample, and polydisperse index (PDI) The tests were performed in triplicate analysis.

#### 2.4.2. Encapsulation Efficiency

The encapsulation efficiency (EE) of ghrelin-loaded liposomes was determined using the Braford method [21]. First, unencapsulated ghrelin was removed from liposomes by centrifuge for 30 min in 15,000 rpm (Sorvall ST 16—Thermo Fisher Scientific, Waltham, MA, USA) at 5 °C. Next, the supernatant containing free ghrelin was separated and liposomes sedimented. Additionally, a PBS pH 5.9 solution containing ghrelin was centrifuged to confirm the non-sedimentation of free ghrelin. Next, the liposomes were dissolved in Bradford reagents, and the ghrelin was released and quantified. The encapsulation efficiency (EE) of ghrelin in coated liposomes was calculated using the following Equation (1):

EE% = (Wt/Wi) × 100%
(1)

where Wt is the total amount of ghrelin in the liposome suspension and Wi is the total quantity of ghrelin added initially during preparation. The construction of the analytical curve for proteins was carried out between concentrations of 1.5–150 µg.mL^−1^. The samples were analyzed by UV–visible spectroscopy using a wavelength of 595 nm.

### 2.5. Selection of the Formulation

The restrictions establishing size targets (minimum), PDI (minimum), zeta potential (maximum), and EE (maximum) were applied to the dependent variables, and the software selected the optimal formulation with the highest statistical desirability factor by a numerical technique.

### 2.6. Stability Studies

The optimized formulations selected were analyzed for the following properties: particle size, PDI, zeta potential, and EE as a function of time (zero, 7, 15, 30, and 60 days), stored in refrigeration (5—8 °C). The methods used in the particle size, PDI, and zeta potential analyses were determined as described in Section 2.4.1. The EE was determined by method of Bradford [21] after ultracentrifugation, as described in Section 2.4.2. The statistical comparisons between the different formulations were carried out through the ANOVA tool ANOVA with Tukey’s post-test and 95% confidence interval and significance with *p* < 0.05.

### 2.7. Transmission Electron Cryo-Microscopy

For freezing in amorphous ice, the samples were prepared in carbon grids (Lacey Carbon Type A 300 mesh copper grids, Ted Pella Inc., Redding, CA, USA), which were previously submitted to the glow discharge procedure in an easy-Glow (PELCO—Redding, CA, USA) equipment, with a 15 mA current, negative charge, and 25 s of discharge.

The freezing in amorphous ice (cryo-preparation) was performed using a Vitrobot Mark IV robotic equipment (FEI, Eindhoven, The Netherlands). To prepare the samples, 3 µL were dripped on the negatively charged grids and allowed to settle for 20 s at a temperature of 22 °C and relative humidity of 100%. The grids containing the sample were blotted for 2.5 s with a force of 5, and waited 20 s. with a single blot and vitrified rapidly plunging into liquid ethane at −145 °C. After immersion in liquid ethane, the grids were kept in liquid nitrogen at −196 °C.

The electron microscopy analyses were then performed under the transmission electron microscope (TEM-1400 PLUS, JEOL, Tokyo, Japan), equipped with lanthanum hexaboride filament (LaB6), operating at 120 kV; the microscope was equipped with a GATAN INC CCD camera. (MultiScan 794, Pleasanton, CA, USA) with a resolution of 1 k × 1 k pixels for digital image acquisition. The grids containing the sample were kept at −173 °C in the microscope chamber throughout the analysis period. The digital micrograph software (Gatan Inc., Pleasanton, CA, USA) was used to analyze the results.

### 2.8. Preparation of the Porcine Mucosa

The model membrane used was the porcine nasal mucosa, as described by Osth et al. [22]. The heads of the pigs were donated by the slaughterhouse (Frigorífico Angelelli. Piracicaba, Brazil). Briefly, the snout was separated from the animal and opened to expose the shell. The mucosa covering the ventral nasal shell (cavity mucosa) was carefully removed from the cartilages using forceps and a scalpel. The mucosa was kept in 0.9% (m/v) sodium chloride solution and frozen. On the day of the experiment, the membranes were thawed and kept immersed in refrigerated saline solution.

### 2.9. Transmucosal Permeability

The study was performed on Franz’s cell diffusion using porcine nasal mucosa, as described by Carvalho [23]. The porcine nasal mucosa (1.76 cm^2^) was placed between the donor and the receptor chambers of a Franz diffusion cell. The 5 mL aliquots of the liposomal formulations containing ghrelin and free ghrelin in PBS pH 5.9 solution were placed in the donor compartment and 15 mL of PBS pH 5.9 was added in the receptor compartment. Additionally, a PBS pH 5.9 solution was tested to confirm the non-interference of possible membrane proteins in the Bradford reagent reading. The receiving chamber is jacketed to maintain the circulation and temperature of the fluid from the thermostatic bath pump. The total test time was 12 h (2 h interval), and 2 mL of the receptor fluid was collected and replaced by an equal volume of fresh receptor solution. The quantitative determination of ghrelin permeated per unit area (µg/cm^2^) was analyzed by the Bradford method [21].

### 2.10. Mucoadhesion Measurement by Tensile Strength Method

The mucoadhesive properties of the selected formulation were evaluated using TA. XT Plus Texture Analyzer (Stable Micro System, Morley, UK). The porcine nasal mucosa was fixed with double-sided tape and attached to the lower end of the analytical probe. A sample of 2 mL was transferred to the sample holder and kept in a water bath with temperature adjusted to 37 °C. The mucosa fixed in the probe was compressed onto the samples with a force of 0.1 N, directed in the apical–basal direction. The contact time of the mucosa with the sample was standardized at 300 s. The probe was removed from the sample surface at a constant speed of 1.0 mm. s^−1^ in the basal–apical direction. The force required to highlight the mucosa from each formulation’s surface was determined from a time-to-force ratio. Measurements were performed in triplicate.

## 3. Results

### 3.1. Quality by Desing Approach

The implementation of the QbD concept involves all the factors that may have an impact on the quality of the final product. The QbD elements seek to direct the objectives of the process, and its tools aim to monitor quality through systematic control of the variables involved in the process [17,20]. The first element of the QTPP is represented by the set of attributes of the product related to the characteristics necessary to achieve the therapeutic objectives [20]. Thus, the QTPPs of the ghrelin-carrier liposomal product were identified and targets and justifications defined (Table 1).

The nose-to-brain delivery systems show a potential alternative to ghrelin administration; however, it suffers limitations such as mucociliary clearance, enzymatic degradation, and limited permeation of large molecules. Therefore, identified the CMAs and CPPs parameters are essential to developing liposome formulation able to (i) protect ghrelin, (ii) increase its residence in the nasal cavity, and (iii) improve its pass through the olfactory epithelium. The critical material attributes (CMAs) refer to the source of variation in the manufacturing process, while the critical process parameters (CPPs) refer to process parameters that plays a significant role in the quality of the product [17]. In this study, liposomal size (Y1), polydispersion index (Y2), zeta potential (Y3), and encapsulation efficiency (Y4) were identified as CQAs of the final product.

**Figure 1:**
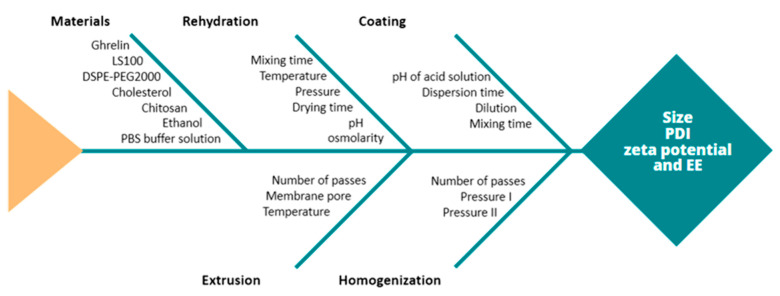
Ishikawa diagram with critical material attributes (CMA) and critical process parameters (CPP) identified and risk analysis in the production of peptide carrier liposomes. (LS100) Soy phosphatidylcholine; (DSPE-PEG2000) sodium salt of 1,2-diestearoyl-sn-glicero-3-phosphoethanolamine-N-[carboxi-(polyethylene glycol) 2000; (PBS) phosphate-buffered saline; (PDI) polydispersion index; (zeta) zeta potential; (EE) encapsulation efficiency.

The application of a quality management visualization tool, such as a fishbone diagram or effect diagram (Ishikawa), is always useful for the identification of the CMAs and the CPPs of the aimed liposomal product [32]. Figure 1 shows the Ishikawa diagram with CMA and CPP identified and risk analysis in the production of peptide carrier liposomes and selecting the most influential CQAs. Table 2 presents the risk analysis related to CMAs and CPPs with estimated impact on quality critical attributes to ghrelin concentration ([ghrl]—X1), chitosan concentration ([ch]—X2), and two CPPs: coating time (X3) and post-formation processing method (X4).

In a previous study, it was found that among CMAs the ghrelin concentration had the highest influence in efficiency encapsulation (EE), and medium influence in particle size and particle size distribution (PDI). The chitosan concentration had the highest influence on particle size and zeta potential. The CPP with the highest influence in CQAs was the post-formation method of ghrelin-liposome, influencing particle size and PDI.

The software allows a graphical interpretation of the results and effects of each parameter in the QbD formulation. The results of the risk analysis gave the basis of the factorial experimental design (DoE), which was built up based on variations of the CMAs and CPPs, as shown in Table 3. The details of the 24 formulations obtained by DoE and its CQAs values are presented and Table 4.

### 3.2. Response Analysis for QbD Optimization Approach

Polynomial equations were generated to describe the individual main effects and interaction effects of the independent variables on the dependent factors individually. Equation (2) is generally structured as follows:

CQA = a + b ∗ [ghrl] + c ∗ [ch] + d ∗ time + e ∗ post-f(2)

where, CQA (critical quality attribute); a (characteristic constant of each CQA); b (ghrelin concentration coefficient); c (chitosan concentration coefficient); d (coating time coefficient); e (post formation processing coefficient); [ghrl] ghrelin concentration; [ch] chitosan concentration; time [coating time]; and post-f [post formation processing].

When adjusting the resulting response data for several models (linear, two-factor interaction, quadratic, and cubic) it was found that the responses for size, zeta potential, and EE were better explained by the quadratic model, while the PDI was better explained by the linear model. Table 5 presents the summaries of the responses, with maximum and minimum values, mean and standard deviation, and the selected model.

#### 3.2.1. Size

The mean size of nanoparticles is an important parameter for the nose-to-brain route. According to the literature, when nanoparticles have a diameter of less than 20 nm can achieve extracellular transport from the nasal cavity to the brain [33]. While nanoparticles larger than about 20 nm are thought to pass by transcellular route (apical to basolateral transport through epithelial cell) [33]. Another possibility, since the average diameter of olfactory axon is about 200 nm (for two-month-old rabbits), the particles of sufficiently small sizes may translocate by the olfactory neural pathway [34].

One of the crucial steps to produce liposomes with sizes and uniformity suitable, for their purpose, is the post-formation processing methods (e.g., sonication, extrusion, and homogenization). In the extrusion method, the size reduction is managed under mild and more reproducible conditions compared to those discussed above. In both cases, even under minimal pressure conditions, homogenization causes a variation in liposome size. However, the high pressure and homogenizer in cycles on high pressures are related as an ideal method for large production scales [35].

**Figure 2:**
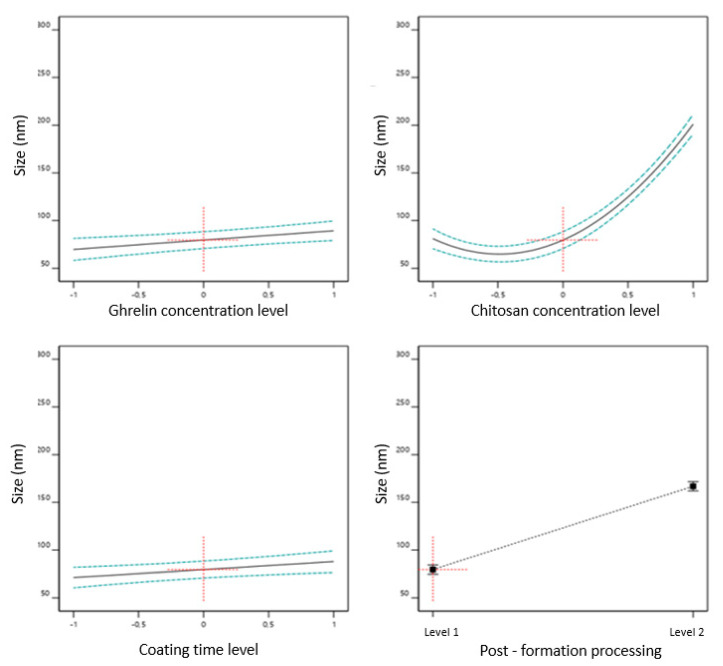
Impact prediction profile of effect of the critical material attributes (CMAs) and critical process parameters (CPPs) on liposome size.

Figure 2 shows the impact prediction profile of (i) ghrelin concentration, (ii) chitosan concentration, (iii) coating time, and (iv) post-formation processing method on liposomal size. The coded equation (Equation (3)) was used to identify the relative impact of each factor by comparing the factor coefficients, that is, the higher value, the greater is the effect of that factor CMAs/CPPs (shown in Figure 2) on the response. A negative effect shows an inverse relationship between a factor and the response [36].

Size = 123.196 + 9.76769 ∗ [ghrl] + 59.9395 ∗ [ch] + 8.26868 ∗ time + 43.6249 ∗ postf + 61.4275 ∗ [ch]2(3)

The prediction profile (Figure 2) indicates that the liposome size increased, sequentially, with the increasing chitosan concentrations (coefficient: +59.9395), post-formation processing (coefficient: +43.6249), ghrelin concentrations (coefficient: +9.7676), and increasing coating time (coefficient: +8.2686). The minimum and maximum values to liposome size can be observed in Table 4. The different chitosan concentrations produced liposomes ranging from 70.25 (0.1% *w*/*w*) to 299.70 nm (0.5% *w*/*w*), with a mean 160.81 ± 72.25 nm. While the high-pressure homogenization method, ranged from 70.25 to 212.14 nm, with a mean of 112.33 ± 48.77nm; the extrusion method varied from 135.97 to 299.70 nm, with a mean of 201.85 ± 64.87 nm.

Salade et al. [14] developed a ghrelin-containing formulation based on liposomes coated with chitosan intended for the nose–brain delivery. The formulations were composed of Lipoid S100, cholesterol with di-hexadecyl phosphate (DHDP) or 1,2-dioleoyl-3-trimethylammonium-propane (DOTAP), and chitosan (1% *w*/*w*) as the coating. Their results showed an increase (21.6%) in average sizes compared to uncoated liposomes, reinforcing the influence of this variable on liposomal size.

Najlah et al., (2019), developed a niosomes nanodispersions manufactured using high-pressure homogenization following the hydration of proniosomes. Using beclomethasone dipropionate (BDP) as a model drug, the characteristics of the homogenized niosomes were compared with vesicles prepared via the conventional approach of probe-sonication. The niosomes generated using high pressure homogenization showed particles with the size 209.20 ± 21.40 nm, while probe-sonicated vesicles showed 236.50 ± 13.00 nm. An important factor observed was the high-pressure homogenization that produced small size niosomes using a short single-step size reduction procedure (6 min to perform 12 cycles) as compared with the time-consuming process of sonication of 418 min [37]. Ong et al. (2016) studied the efficiency of different techniques (extrusion, sonication, ultrasonication, homogenization, and freeze–thaw-sonication) used for nanosizing liposomes. Further, to evaluate the effect of process parameters of extrusion techniques on the size and size distribution of the resultant liposomes. They concluded that the extrusion technique produced the smallest and most homogenous liposomes, followed by freeze–thaw-sonication, ultrasonication, sonication, and homogenization [38].

#### 3.2.2. Polydispersity Index

The PDI is a representation of the distribution of size populations in each sample. The PDI’s value ranges from 0.0 (for a perfectly uniform sample with respect to the particle size) to 1.0 (for a highly polydisperse sample with multiple particle size populations). In drug administration applications using lipid-based carriers, such as liposome and nanoliposome formulations, a PDI of 0.300 or below is considered acceptable and indicates a homogeneous population of phospholipid vesicles [29]. The FDA “Guidance for Industry” on liposomal drugs [39] emphasizes the importance of size and size distribution as CQAs, and essential components of stability studies of these products.

**Figure 3:**
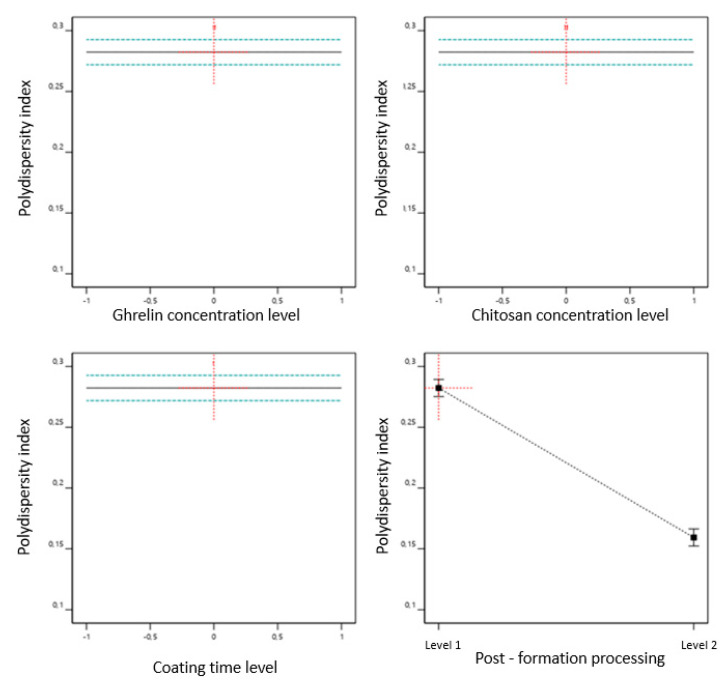
Effect of critical material attributes (CMAs) and critical process parameters (CPPs) on the polydispersity index.

Figure 3 shows the effect of CMAs and CPPs on the PDI. The variable that influenced the PDI was the post-formation processing. The mean PDI for extrusion ranged from 0.130 to 0.194, with a mean of 0.159 ± 0.019. While for high pressure homogenization it varied from 0.263 to 0.298, with a mean of 0.282 ± 0.012. Thus, the extruded samples showed more uniformity in relation to the particle size, but the samples treated with homogenizer obtained a size distribution within acceptable limits (less than 0.300). To know the influence of quantitative form of the post formation processing in the PDI, the coefficient was determined by Equation (4).

PDI = 0.22079 − 0.0614825 ∗ postf(4)

The coefficient value obtained was −0.0614, this suggests that comparing the levels of the categorical variable (post-formation processing), extrusion (level 2) results in PDI lower than high-pressure homogenization (level 1).

#### 3.2.3. Zeta Potential

The zeta potential values provide information on the character and functionality of the surface and have great significance in the liposomes field [35]. The zeta potential has an important role in controlling the stability of liposomes by the repulsive energy barrier opposing the aggregation of dispersed liposomes in buffer solutions [40,41]. If all particles in the suspension have a large negative or positive zeta potential, they will repel each other, and there will be no tendency for particles to aggregate [42]. The potential zeta of the uncoated chitosan liposome was negative at −29.90 ± 7.76 mV while the potential zeta of the coated liposome was positive at 50.3 ± 1.46 mV. The increase in zeta potential was attributed to cationic groups of chitosan adsorbed on the liposomal surface. This occurs to electrostatic interactions between the negatively charged phospholipids and the positive charges of primary amino groups of chitosan, which help to increase the stability of liposomes and prevent leakage from them [43]. The mix of cholesterol (neutral lipid) and negatively charged phospholipids like phosphatidylethanolamine are the responsible negative zeta potential of uncoated liposomes. Therefore, the main interaction at play between the positive amino groups of chitosan and oppositely charged lecithin phospholipids can be argued to be the final electrostatic charge.

**Figure 4:**
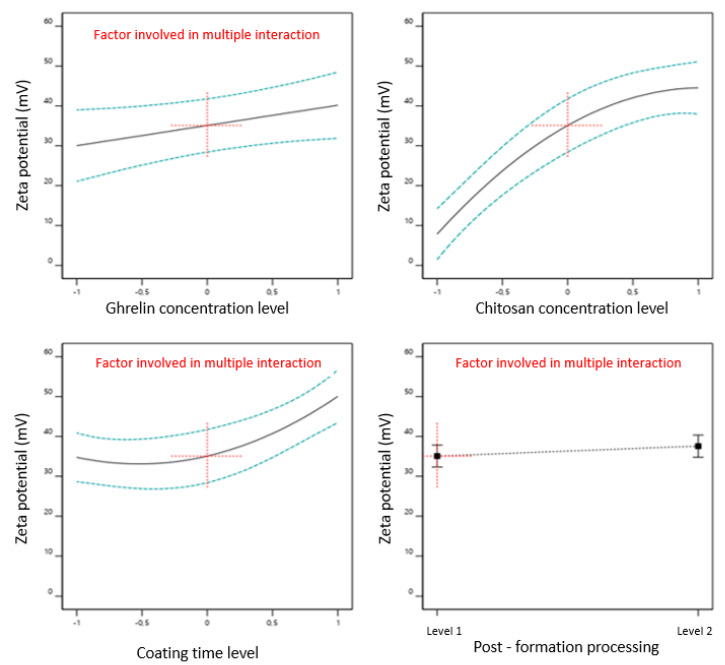
Effect of critical material attributes (CMAs) and critical process parameters (CPPs) on zeta potential.

Figure 4 showed that the four factors studied (ghrelin concentration, chitosan concentration, time of coating, and post-formation processing method) had an impact on the zeta potential, and Equation (5) represents the effect of factors on the potential zeta of liposomes:

Zeta potential = 36.334 + 1.4574 ∗ [ghrl] + 18.3276 ∗ [ch] + 7.62214 ∗ time + 1.24225 ∗ postf + 4.82753 ∗ [ghrl] ∗ time − 3.60853 ∗ [ghrl] ∗ postf − 8.90764 ∗ [ch]2 + 7.3319 ∗ time2(5)

**Figure 5:**
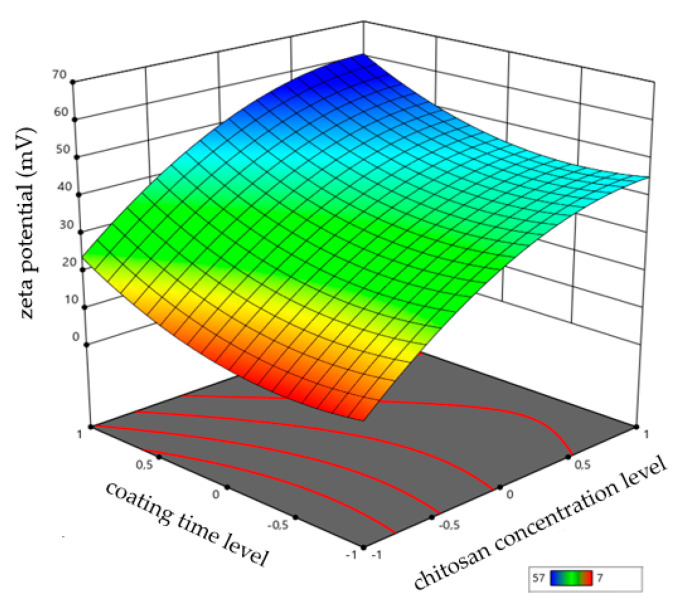
Response surface for the zeta potential of liposomes as a function of chitosan concentration and coating time.

The interactions between the factors are also shown in the equation, showing the interaction coefficients between CMAs/CPPs. The value of the coefficient of chitosan concentration (+18.3276) indicates that this was the variable that most influenced the zeta potential of the liposomes, followed by the coating time, with a coefficient of +7.62214. The simultaneous influence of chitosan concentration and coating time on the zeta potential of liposomes is shown in the surface response of Figure 5.

#### 3.2.4. Encapsulation Efficiency

**Figure 6:**
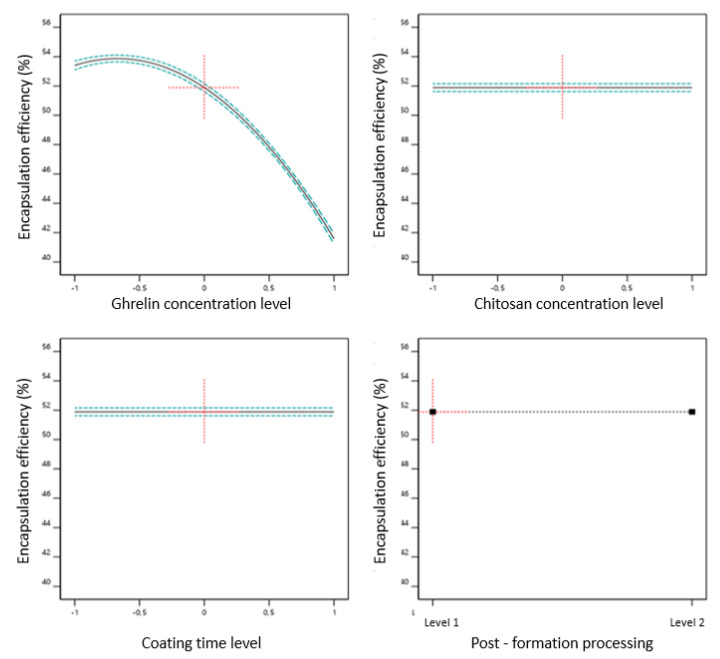
Effect of critical material attributes (CMAs) and critical process parameters (CPPs) on encapsulation efficiency.

The ranged encapsulation efficiency (EE) from 40.9 to 53.9%, with an average of 49.82% ± 4.91% of ghrelin, may be explained as an expected consequence of the positively charged chitosan and positively charged ghrelin competing for the negatively charged phospholipids. Mangoni et al., (2017), developed chitosan-coated liposomes loaded with substance P (neuropeptide of 11 amino acids) and obtained liposomes with an EE of around 66% [43]. Another hypothesis is the hydrophilicity of ghrelin, which can be easily dissolved in the external aqueous phase during the hydration of the lipid film, whose volume is greater than the volume trapped inside the aqueous cavity liposome. Figure 6 shows the CMAs and CPPs influence in EE of ghrelin. It was observed that the ghrelin concentration was the only variable that influenced EE, and the coefficient obtained by Equation (6) shows quantitatively how much this CMA influences EE.

EE = 51.89 – 5.9 ∗ [ghrl] – 4.39 ∗ [ghrl]2(6)

The values of the coefficients in Equation (6) suggest the ghrelin concentration had a negative effect on the encapsulation efficiency, that is, the encapsulation efficiency decreased with the highest concentration of ghrelin.

### 3.3. Selection of the Optimal Formulation QbD-Based

**Figure 7:**
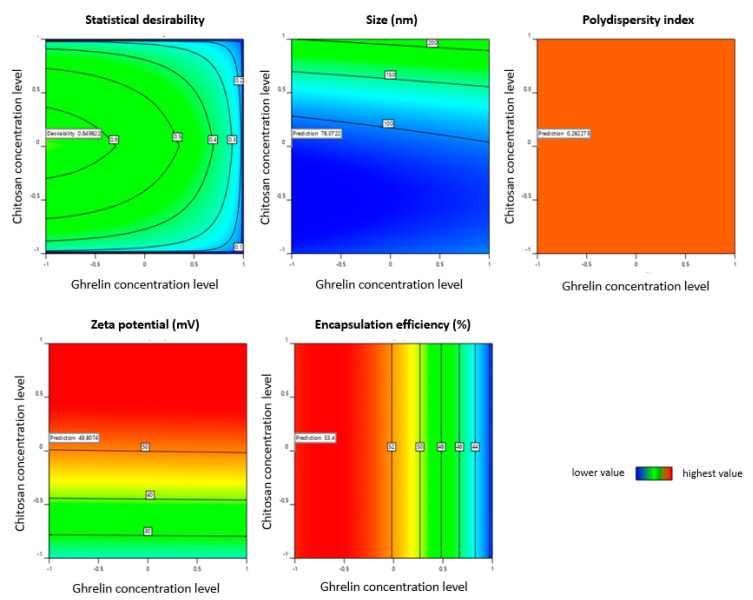
Contour plot for the effect of CMAs variables presented in the highest statistical desirability for the high-pressure homogenization method, establishing the target criteria of size (minimum), polydispersion index (minimum), zeta potential (maximum), and encapsulation efficiency (maximum).

Based on the prediction profile for maximum desirability of the target level of responses, contour plots (Figure 7) were arranged to get the design space and suitable range of the critical factors identified to achieve target responses. The formulation obtained by high-pressure homogenization showed the highest statistical desirability, establishing the target criteria of size (minimum), PDI (minimum), zeta potential (maximum), and EE (maximum).

The liposomes produced according to the parameters predicted by numerical optimization and the actual responses obtained are shown in Table 6. The software analyses suggested that the high-pressure homogenization method will produce a liposome size of 78.07 nm, a PDI of 0.282, a zeta potential of 49.81 mV, and an encapsulation efficiency of 53.40%. While the liposomes obtained showed a size of 72.25 ± 1.46 nm, a PDI of 0.300 ± 0.027, a zeta potential of 50.3 ± 1.46 mV, and an EE of 53.2%. This formulation was named Lhomo. Thus, the selected formulation with the highest statistical desirability factor was that with CMAs factors of 70 µg.mL^−1^ of ghrelin concentration and 0.3% (*w*/*w*) of chitosan; CPPs factors of 24 h coating time, and post-formation processing by high-pressure homogenization.

**Figure 8:**
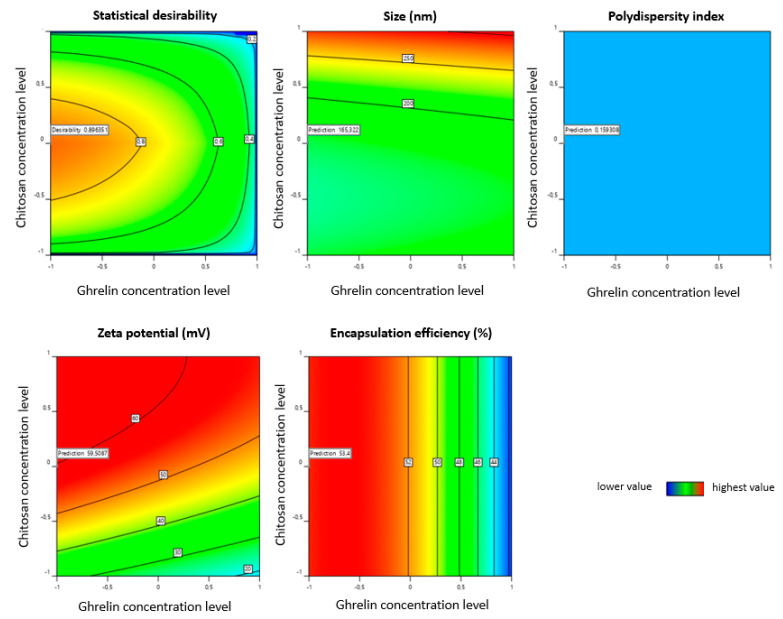
Contour plot for the effect of CMAs variables presented in the highest statistical desirability for the extrusion method, establishing the target criteria of size (minimum), polydispersion index (minimum), zeta potential (maximum), and encapsulation efficiency (maximum).

For comparison, the best results for extruded liposomes were identified through numerical optimization. as shown in contour plots (Figure 8). The software analyses suggested the extrusion method will produce a liposome size of 32 nm, a PDI of 0.159, a zeta potential of 59.5 mV, and an encapsulation efficiency of 52.9% (Table 7). The results obtained for the liposomes produced, according to the parameters predicted by numerical optimization, showed the size of 152.43 ± 0.24 nm, a PDI of 0.159 ± 0.018, a zeta potential of 60.81 ± 6.61 mV, and an EE of 53.6%. This formulation was named Lextru (liposome extrusion).

### 3.4. Stability Studies

For safe and effective use of liposomes, it is critical to establish their stability, which has substantial impacts on liposome storage and further applications. This stability is directly dependent on both formulation and manufacturing method parameters.

Particle aggregation (thermodynamic instability) and sedimentation (kinetic instability) are two essential aspects of colloidal systems instability, as liposomes. The size increase can directly affect the efficacy of liposomes since particle size has been shown to have great impacts on cellular uptake, cytotoxicity, pharmacokinetic profile, and tissue distribution [44].

Cadeo et al. [45] and Doi et al. [46], reported that liposomes modified with PEG (containing resveratrol or oxaliplatin), over a (2 or 12 months, respectively) storage period at 4–8 °C, showed no physical changes, whereas conventional liposomes showed progressive aggregation and precipitation. In addition, the adsorption efficiency for PEG-modified liposomes did not decrease significantly for two months of storage, with no signs of degradation.

The stability of ghrelin-loaded liposomes, on the formulations selected, was examined over a period of 7, 15, 30, and 60 days in triplicate. The results and statistical analyses are shown in Table 8 for liposomes obtained by high-pressure homogenization, and Table 9 for liposomes obtained by extrusion method.

Particle size, PDI, and zeta potential were monitored and did not display any significant change compared to the initial sample (day 1), except for liposome obtained by the extrusion method after 60 days.

EE varied statistically during the 60 days period, but the time starts (t = 0) and the final time (t = 60 days) had no statistical difference. The variation between the times may suggest that the formulations present a period of accommodation of the drug in the liposome. Moreover, there was no significant change in the appearance of liposomes. Thus, the developed formulation from selected screened factors was found to be stable with respect to the selected responses.

### 3.5. Cryo-Transmission Electron Microscopy

Cryo-transmission electron microscopy (cryo-TEM) is an important characterization method for the size and shape of liposomes measurement. It provides a directly visualize individual of internal architecture without structural disturbance and an accurate determination of liposome size [47].

**Figure 9:**
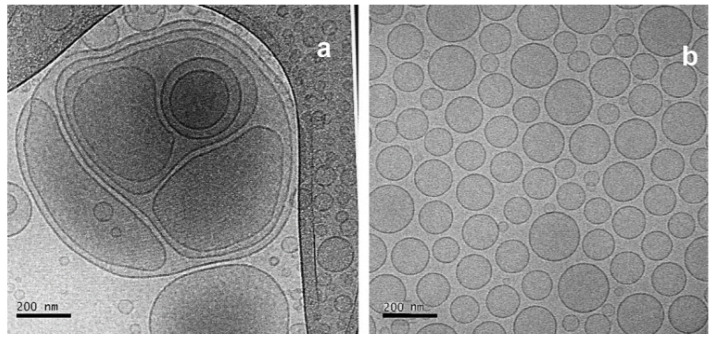
Micrographs obtained by cryo-TEM from liposomes from pre and post extrusion processing. (**a**): Before extrusion processing; (**b**): post extrusion processing.

Figure 9 shows images of the liposomes generated by the cryo-TEM system before and after the extrusion method; both kept the structures close to the bilayer, spaced by a free internal structure maintaining structural integrity. The measurement of the size of the liposomes (150 nm) was like the average recorded by the DLS technique (Table 7). Figure 9a shows multilamellar liposome before extrusion processing and small and homogeneous liposomes post extrusion process (Figure 9b).

### 3.6. Mucosadhesive Property

The detachment forces between polymeric films or tablets and animal mucosae are frequently determined to evaluate the mucoadhesion strength of polymers. The mucoadhesion mechanism has two stages, (i) the contact (wetting) stage followed by the (ii) consolidation stage (the establishment of the adhesive interactions). The first stage is characterized by the contact between the mucoadhesive system and the mucus membrane, with spreading and swelling of the formulation. While in the (ii) consolidation step, the mucoadhesive system is activated by the presence of moisture, allowing the mucoadhesive molecules to break free and to link up by weak van der Waals and hydrogen bonds [48,49].

Furthermore, there are two theories explaining the consolidation step: the diffusion theory and the dehydration theory. In diffusion theory, the mucoadhesive molecules and the glycoproteins of the mucus mutually interact by means of the interpenetration of their chains and the building of secondary bonds. Additionally, in dehydration theory, a mucoadhesive system can jellify in an aqueous environment, due to the difference in concentration gradient that draws the water into the formulation until the osmotic balance is reached [48,49].

Chitosan and its derivatives have been shown to be active in enhancing intranasal drug absorption due to their excellent mucoadhesive properties. The interactions between mucin and chitosan occur primarily due to electrostatic binding supported by forces such as hydrogen bonding and hydrophobic association [50].

The results for maximum separation force (mN) and adhesion work (N/s) for Lhomo, uncoated Lhomo, Lextru, and uncoated Lextru formulation are shown in Table 10. The mucosal-adhesive properties of chitosan-coated formulations were significantly higher than uncoated formulations in the maximum separation force (mucoadhesion) and adhesion work parameters.

These analyses confirm the effect of the coating seen in the zeta potential analyses. The potential zeta of the uncoated chitosan liposome was negative of 29.90 ± 7.76 mV and the potential zeta of the coated liposome was positive for 50.3 ± 1.46 mV. The correlation between zeta potential and mucoadhesion might be explained by the fact that the interactions by electrostatic interactions between protonated amino groups (NH3^+^ of mucoadhesive polymer and negatively charged groups, like carboxylate (COO^−^ or sulphonate (SO_3_^−^) groups, of protein-carbohydrate chains. Moreover, some hydrophobic contributions can be also involved in the interactions of chitosan with mucin [51]. Thus, the capacity of chitosan-coated liposomes to interact with the negatively charged mucosal surface is important to prolong the contact time of liposomes. Additionally, this feature when added to the capacity of chitosan in opening the tight junctions between mucosal cells may improve the ghrelin bioavailability.

### 3.7. Transmucosal Permeation

**Figure 10:**
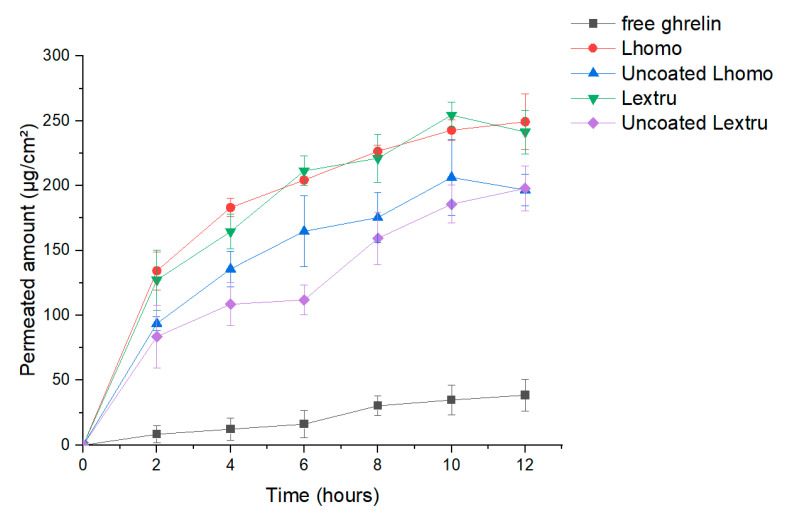
Ex-vivo ghrelin release profiles through of the porcine nasal mucosa: ghrelin free, Lhomo, uncoated Lhomo, Lextru, and uncoated Lextru formulations.

The porcine nasal mucosa is morphologically like the human nasal mucosa. Ex-vivo release studies were carried out to analyze the effect of permeation of free ghrelin, coating and uncoated formulations through the nasal mucosa of porcine. The permeability of free ghrelin through the mucosa, as shown in Figure 10, was lower and slower than liposomal carrier formulations.

Free ghrelin exhibited a maximum permeation rate of 11.0% in 12 h, while the permeation profile of uncoated liposomes obtained by extrusion and high-pressure homogenization showed a permeation rate of 56.45% and 56.20% of ghrelin, respectively. The chitosan coated liposome obtained by extrusion and high-pressure homogenization showed a permeation rate of 71.23% and 68.97% of ghrelin, respectively. The analysis of results confirmed the higher permeation of liposomal formulations when compared to free ghrelin. This behavior can be attributed to the characteristics of the liposomes that affect the permeability of the epithelial cell layer interacting with the phospholipid layer and the outer layer of the mucosa.

Another point observed was the greater permeation of formulations coated with chitosan when compared to liposomal formulations without the coating. These results are directly correlated with the mucoadhesion study, shown previously (Section 3.7). Where, the amino groups bond ionically with sulfonic acid of the mucus and the amino and hydroxyl groups form hydrogen bonds with the mucus, leading to the reversible opening of the tissue junction, and the permeation of substances through the nasal epithelium is increased [52]. The impact of chitosan on permeation was evaluated to its potentiating effect on the permeation of the peptide through the layers of the nasal mucosa, as also observed in other studies [14,53].

## 4. Conclusions

The QbD-based approach was successfully adapted in the initial phase of developing a ghrelin carrier liposomal formulation. The QTPP elements were defined, and the lipid film hydration method was selected to prepare the desired liposomes. Process-related CPPs/CMAs and CQAs were determined. The CQAs were influenced by the most critical elements of the CMAs and CPPs and formed the pattern of the experimental design; thus, the liposome preparation was focused on the most critical parameters. It was possible to establish the process parameters that influence the particle size, PDI, zeta potential, and EE. Among the formulation factors, chitosan concentration was the variable that most influenced the size of liposome, followed by post-formation processing. While ghrelin concentration was the main parameter influencing EE, the EE decreased with the highest ghrelin concentration. Liposomes treated by extrusion showed a larger size concerning liposomes treated by high-pressure homogenization; however, both obtained a PDI within acceptable limits (<0.300). Analyses of particle size, PDI, zeta potential, and EE results showed that these parameters remained stable for 60 days. The zeta potential higher than ± 30 mV stabilized the dispersion of liposomes blocking the aggregation of structures due to electrostatic repulsion. The chitosan coating effectively obtained a positive result of zeta potential and mucoadhesion in an ex vivo study, aiming to overcome mucociliary activity limitation. Formulations with liposomes as nanocarriers improved permeation through the nasal mucosa and are also essential for effective protection against enzymatic degradation, especially for biotherapeutics. Furthermore, chitosan coating has also positively influenced ghrelin permeation through the porcine mucosa in an ex vivo study. The characteristics related to ghrelin suggest that it may be able to reach the brain tissue via nasal administration. The results confirmed that the QbD approach could improve the development of liposomal formulation, lead to an effective product preparation process, and help to optimize and rationalize liposomal development. Furthermore, future ex vitro and in vivo studies are necessary to confirm this release profile; however, the liposomal formulations were designed with the necessary characteristics.

## Figures and Tables

**Table 1:** Quality target product profile (QTPP) of the ghrelin-carrier liposomal product.

QTPP Element	Target	Justification
Indication	Cachexia treatment	One of the proposed mechanisms of action for ghrelin assumes that this hormone regulates metabolism by activating orexigenic neuronal circuits, such as the central melanocortin system [24].
Administration route	Intranasal	The intranasal route offers direct access to the central nervous system [25].
Dosage form	Liquid suspension	The use of the liquid form of the liposomal formulation offers a comfortable method for the administration of the drug in the nasal cavity [26].
Particle size (nm)	70–400	The ideal particle size range for intranasal administration if the target is specifically nose-to-brain delivery is 70–400 nm [27,28].
Polydispersion index	<0.3	In applications of nanoliposome administration, an PDI of 0.3 and below is considered acceptable and indicates a homogeneous population of phospholipid vesicles [29].
Zeta potential (mV)	> (+) 20	Cationic nanocarriers are more efficient vehicles for delivering medicines to the brain due to electrostatic interactions [30].
pH	4.5–6.5	The optimum pH of the preparation adjusts to the normal pH of the intranasal environment. The appropriate pH value ensures comfort during application and determines the quality and in vivo effectiveness of the product [31].
Stability	60 days	The stability of the size of the prepared liposomes, uniformity and surface load is linked to the effectiveness and quality of the preparation

**Table 2:** Risk analysis related to critical material attributes (CMAs) and critical process parameters (CPPs) with estimated impact on quality critical attributes (CQAs).

	Variables	Estimated Impact on CQAs				Appropriate Range
		Particle Size	PDI	Zeta Potential	EE	
CMAs	[ghrl]—X1	M	M	L	H	70–140 µg.mL^−1^
	[ch]—X2	H	M	H	L	0.1–0.5% (*w*/*w*)
	CT—X3	M	L	H	L	2–24 h
CPPs	Post-FP—X4	H	H	L	L	High-pressure homogenization or extrusion

(PDI) polydispersion index; (zeta) zeta potential; (EE) encapsulation efficiency; (H) high; (M) medium; (L) low. [ghrl] ghrelin concentration; [ch] chitosan concentration; [CT] coating time; (Post-FP) post-formation process; (X1-X4) variables X1–X4.

**Table 3:** Design of experiments (DoE) variables evaluated for the development of liposomal formulations.

CMAs and CPPs	Level					
	Low (−1)		Medium (0)		High (+1)	
Ghrelin concentration (µg. mL^−1^)	70		105		140	
Chitosan concentration (% *w*/*w*)	0.1		0.3		0.5	
Coating time (hours)	2		8		24	
	Level 1			Level 2		
Post—formation processing	Homogenization			Extrusion		
CQAs	Objective					
Liposomal size (nm)	Minimum					
Polydispersion index	Minimum					
Zeta potential (mV)	Maximum					
Encapsulation efficiency (%)	Maximum					

(CMA) Critical material attributes; (CPP) critical process parameters, and (CQA) quality critical attributes.

**Table 4:** Details of the evaluated variables of design of experiments (DoE) and its respective results, for the development of liposomal formulations.

Run	[ghrl] (µg/mL)	[ch] (% *w*/*w*)	Time (Hours)	Post-f	Size (nm)	PDI	Zeta (mv)	EE (%)
1	70	0.5	2	homo.	170.72	0.294	42.70	52.80
2	105	0.5	8	homo.	181.54	0.277	48.30	52.50
3	70	0.1	2	homo.	70.25	0.272	10.88	53.70
4	140	0.3	24	homo.	91.87	0.263	51.30	41.70
5	140	0.1	8	extru.	181.40	0.147	7.64	41.90
6	105	0.5	2	extru.	284.76	0.164	48.30	51.90
7	70	0.3	2	extru.	135.97	0.157	39.90	53.40
8	105	0.3	8	extru.	145.76	0.18	47.70	52.30
9	105	0.5	24	extru.	294.34	0.152	56.40	51.50
10	70	0.1	24	extru.	160.45	0.173	33.70	53.50
11	105	0.1	2	homo.	74.57	0.286	7.20	51.90
12	140	0.5	8	extru	299.70	0.186	42.40	41.60
13	70	0.1	8	extru.	157.34	0.194	8.77	53.90
14	105	0.1	8	homo.	84.24	0.289	9.63	51.40
15	105	0.5	8	homo.	212.14	0.274	44.60	51.80
16	140	0.3	24	homo.	92.77	0.289	50.40	41.80
17	105	0.1	8	homo.	83.82	0.266	7.90	51.90
18	105	0.3	8	extru.	173.34	0.15	36.3	51.70
19	70	0.3	24	homo.	93.90	0.298	52.8	52.40
20	140	0.3	2	homo.	79.76	0.297	40.9	40.90
21	105	0.3	8	extru.	170.29	0.13	35.6	52.00
22	140	0.3	2	extru.	179.55	0.143	41.5	41.70
23	70	0.1	2	extru.	146.79	0.137	8.77	53.70
24	70	0.5	8	extru.	294.33	0.158	43.3	53.80

[ghrl]: ghrelin concentration; [ch]: chitosan concentration; (post-f) post formation processing; (PDI) polydispersion index; (zeta) zeta potential; (EE) encapsulation efficiency; (homo) high pressure homogenization; (extru) extrusion.

**Table 5:** Summaries of responses, with maximum and minimum values, mean and standard deviation (SD), and statistical model selected.

	Minimum	Maximum	Average	SD	Model
Liposomal size (nm)	70.25	299.7	160.82	±73.25	Quadratic
Polydispersion index	0.13	0.29	0.216	±0.065	Linear
Zeta potential (mV)	7.20	56.40	34.04	±17.46	Quadratic
Encapsulation efficiency (%)	40.90	53.90	49.82	±4.91	Quadratic

**Table 6:** Expected responses by numerical optimization and actual responses of liposomes produced by high-pressure homogenization method according to the optimized parameters.

	Predicted	Real (M ± SD)
Size (nm)	78.07	72.25 ± 1.46
PDI	0.282	0.30 ± 0.02
Zeta (mV)	49.80	50.30 ± 1.46
EE (%)	53.40	53.20 ± 0.80

**Table 7:** Expected responses by numerical optimization and actual responses of liposomes produced by extrusion method according to the optimized parameters (means ± standard deviation).

Parameters	Predicted	Real (M ± SD
Size (nm)	165.3	152.43 ± 0.24
PDI	0.16	0.159 ± 0.018
Zeta (mV)	59.5	60.81 ± 6.61
EE (%)	53.4	53.6 ± 0.7

**Table 8:** Stability of the Lhomo formulation over time.

Time (days)	Liposomal Size (nm)	PDI	Zeta Potential (mV)	EE (%)
0	72.3 ± 1.4a	0.3 ± 0.027a	50.4 ± 1.46a	53.5 ± 0.3c
7	72.7 ± 2.4a	0.305 ± 0.034a	50.7 ± 1.43a	55.8 ± 0.17a
15	72.3 ± 1.66a	0.282 ± 0.014a	49.9 ± 1.33a	54.6 ± 0.26b
30	72.3 ± 1.12a	0.303 ± 0.082a	50.4 ± 2.45a	53.6 ± 0.2c
60	72.3 ± 1.51a	0.256 ± 0.009a	50.6 ± 1.76a	53.4 ± 0.17c

The same letters (for the same analysis) indicate that there is no significant difference between the means of the values (*p* > 0.05) (*n* = 3). The other results are statistically different. (PDI) Polydispersion index; (zeta) zeta potential; (EE) encapsulation efficiency.

**Table 9:** Stability of the Lextru formulation over time.

Time (days)	Liposomal Size (nm)	PDI	Zeta Potential (mV)	EE (%)
0	152.4 ± 0.2 a	0.159 ± 0.018a	60.9 ± 6.61a	52.9 ± 0.17b
7	153.3 ± 0.16a	0.183 ± 0.023a	60.2 ± 7.72a	52.6 ± 0.3a
15	152.2 ± 0.15a	0.161 ± 0.083a	60.8 ± 5.83a	52.4 ± 0.26c
30	152.4 ± 0.23a	0.183 ± 0.011a	60.8 ± 4.84a	51.9 ± 0.2 a
60	150.4 ± 0.29b	0.173 ± 0.016a	50.9 ± 6.39a	51.7 ± 0.17ab

The same letters (for the same analysis) indicate that there is no significant difference between the means of the values (*p* > 0.05) (*n* = 3). The other results are statistically different. (PDI) Polydispersion index; (zeta) zeta potential; (EE) encapsulation efficiency.

**Table 10:** Result of mucoadhesion (maximum separation force) and work adhesion of liposome coated and uncoated liposome formulations with swine mucosa. Results are expressed as mean ± standard deviation.

Formulations	Maximum Separation Force (mN)	Work of Adhesion (N/s)
Lhomo	84.19 ± 5.64 ^a^	2.84 ± 0.23 ^a^
Uncoated Lhomo	45.48 ± 1.59 ^b^	1.78 ± 0.18 ^b^
Lextru	81.23 ± 4.62 ^a^	2.63 ± 0.14 ^a^
Uncoated Lextru	47.3 ± 2.57 ^b^	1.89 ± 0.05 ^b^

The same letters (for the same analysis) indicate that there is no significant difference between the means of the values (*p* > 0.05) (*n* = 3). The other results are statistically different. (Lhomo) Liposome after homogenization; (Lextru) liposome after extrusion.
